# Bioactive Carboxymethyl Cellulose (CMC)-Based Films Modified with Melanin and Silver Nanoparticles (AgNPs)—The Effect of the Degree of CMC Substitution on the In Situ Synthesis of AgNPs and Films’ Functional Properties

**DOI:** 10.3390/ijms232415560

**Published:** 2022-12-08

**Authors:** Szymon Macieja, Bartosz Środa, Beata Zielińska, Swarup Roy, Artur Bartkowiak, Łukasz Łopusiewicz

**Affiliations:** 1 Center of Bioimmobilisation and Innovative Packaging Materials, Faculty of Food Sciences and Fisheries, West Pomeranian University of Technology Szczecin, Janickiego 35, 71-270 Szczecin, Poland; 2 Department of Nanomaterials Physicochemistry, Faculty of Chemical Technology and Engineering, West Pomeranian University of Technology Szczecin, Piastow Ave. 42, 71-065 Szczecin, Poland; 3 School of Bioengineering and Food Technology, Shoolini University, Solan 173229, HP, India

**Keywords:** melanin, carboxymethyl cellulose, bioactive films, silver nanoparticles, antimicrobial, green synthesis

## Abstract

Green synthesis of nanoparticles for use in food packaging or biomedical applications is attracting increasing interest. In this study, the effect of the degree of substitution (0.7, 0.9 and 1.2) of a carboxymethylcellulose polymer matrix on the synthesis and properties of silver nanoparticles using melanin as a reductant was investigated. For this purpose, the mechanical, UV–Vis barrier, crystallinity, morphology, antioxidant and antimicrobial properties of the films were determined, as well as the color and changes in chemical bonds. The degree of substitution effected noticeable changes in the color of the films (the L* parameter was 2.87 ± 0.76, 5.59 ± 1.30 and 13.45 ± 1.11 for CMC 0.7 + Ag, CMC 0.9 + Ag and CMC 1.2 + Ag samples, respectively), the UV–Vis barrier properties (the transmittance at 280 nm was 4.51 ± 0.58, 7.65 ± 0.84 and 7.98 ± 0.75 for CMC 0.7 + Ag, CMC 0.9 + Ag and CMC 1.2 + Ag, respectively) or the antimicrobial properties of the films (the higher the degree of substitution, the better the antimicrobial properties of the silver nanoparticle-modified films). The differences in the properties of films with silver nanoparticles synthesized in situ might be linked to the increasing dispersion of silver nanoparticles as the degree of CMC substitution increases. Potentially, such films could be used in food packaging or biomedical applications.

## 1. Introduction

Food packaging, whether in the form of hard boxes, thin films or edible coatings, is designed to influence the shelf life of the packaged product and at the same time protect it from mechanical damage or microbial contamination [1]. The relationship between food and its packaging and the waste generated is direct and multifactorial. On the one hand, due to lack of or insufficient protection from external factors, food spoils and consequently requires disposal, which is characteristic of developing countries. On the other hand, in developed countries, the amount of waste generated from food packaging (PET and general plastics) accounts for a large share of total waste [2]. The obvious solutions to this problem are to develop better food packaging that provides greater protection from external factors, and to influence consumer choices to reduce the purchase of products that will eventually end up in the trash as a result of excessive consumerism. The European Union aims to reduce the amount of food waste generated by 30% by 2025, and efforts are already underway to introduce legal solutions and educate consumers about rational food sourcing [3,4]. Consumer awareness of recycling waste such as glass, waste paper and plastics is already quite high, but there is still little discussion of the possibility of managing some organic waste (including agro-industrial waste) as a source of, for example, phenolic compounds, fibers, polysaccharides, vitamins, carotenoids, pigments or oils [5].

Plastic, due to its ease of processing and relatively low cost, has become one of the most widely used materials in almost every industry, with global annual production exceeding 360 million tons (as for 2019) [6,7]. Among the waste that is packaging, up to 60% is food packaging. Going forward, about 80% of plastic waste ends up in landfills or contaminating land and water reservoirs. This has negative (both direct and indirect) effects on many ecosystems through plants, fish and other animals to humans [8,9,10]. Another problem with conventional food packaging is that the production and disposal of plastic involves the release of huge amounts of greenhouse gases, which, in addition to those mentioned above, also negatively affects the environment. Therefore, it would be appropriate to strive to replace conventional plastic packaging with biodegradable and renewable biopolymers [11].

Among biopolymers, cellulose is of great interest due to its high availability, non-toxicity, renewability, biodegradability and outstanding physical properties [12,13]. Cellulose-based biocomposites (known as green composites—composites produced using natural fibers) due to those properties, find many applications in, for example, the biomedical industry for: medical implants, biosensors, drug delivery or skin tissue repair, among others [14]. One of its derivatives, carboxymethylcellulose (CMC), is of exceptional interest to researchers working on films and biopolymer-based packaging. It is water-soluble and forms transparent films with high flexibility and gas barrier properties; however, at the same time, it exhibits strong hydrophilicity which is a limiting factor for its wider use [15,16]. The average degree of substitution (the average number of hydroxyl groups in the sugar ring that have been substituted with a carboxymethyl group) of CMC is in the range of 0.4–1.5 [17]. CMC is also used in the food industry as an emulsifier or stabilizer, among other applications. For these purposes, derivatives with a degree of substitution between 0.7 and 1.2 are used. As studies show, a clear relationship can be observed between the molecular weight and degree of substitution of this biopolymer and the properties of the mixture in which it is used [17].

Since the beginning of the 20th century, there has been growing interest from researchers in developing and improving the so-called “green synthesis” of metal nanoparticles. This is dictated by, among other things, lower process costs, elimination/reduction of the use of environmentally toxic chemicals and simpler methods of obtaining these nanoparticles. This is carried out by using enzymes, vitamins, microorganisms, plants and their extracts/compounds contained therein or microwave radiation, among others [18,19,20,21,22,23].

Potential applications of metal nanoparticles and their oxides in electrical devices, biosensors, antimicrobial surfaces, targeted drug delivery systems or solar cells are reported [24,25]. Among the proposed applications, biomedical and packaging applications are the most prominent. Metal nanoparticles and nanocomposites containing them are pointed out as a potential tool against multidrug-resistant microorganisms. Research focuses mainly on the use of nanoparticles of silver, copper and copper oxides, iron oxides, titanium dioxide, zinc oxide or magnesium oxide for this purpose, which is related to the ever-increasing antibiotic resistance among microorganisms [26]. In addition, in recent years, numerous publications have been written on the application of nanomaterials in food packaging. These works have touched, among others, on titanium dioxide, zinc oxide or silicon oxide, where they have influenced the improvement of packaging properties and the extension of storage time of packaged products [27].

Some of the naturally occurring compounds that fit into the premise of green synthesis of metal nanoparticles are melanins. They act as metal salt reducers and further stabilize the nanoparticles (acting as a capping agent—hindering the formation of agglomerates), so that the use of other substances in this process is not required [28]. The literature describes the use of melanins to obtain silver and gold nanoparticles, as well as zinc and copper oxides, among others [28,29]. Among them, silver nanoparticles (AgNPs) appear as most promising for use in nanocomposites. Their antimicrobial properties against major microorganisms in food contamination are fairly well documented [30]. Their antimicrobial activity is largely due to the induction of pores in cell membranes, which is attributed to the interaction of silver with sulfur-containing membrane proteins. Further, silver, once it enters the cell, can affect the genetic material of the microbe by stimulating its condensation, affecting gene expression or inhibiting replication [31,32,33,34]. Studies on modifying alginate-based films with melanin and silver nanoparticles found no cytotoxic effects on human cells in vitro, so it might be suspected that using AgNPs in films is safe and does not cause any harmful effects for human health, but as stated, further extended studies are needed to conclusively confirm these observations [29].

The effect of the degree of substitution on the green synthesis of silver nanoparticles has been previously determined in various studies [35,36,37]. Hebeish et al. found an increase in the dispersion of silver nanoparticles with an increase in the degree of substitution of the CMC (studying CMC with a degree of substitution (DS) between 1.22 and 2.6) up to a certain limiting point (DS = 2.2) after which an increase in the tendency to agglomerate nanoparticles was again observed [37]. Therefore, it can be suspected that melanin as well as CMC will synergistically affect the synthesis of silver nanoparticles, and the degree of substitution will affect changes in their morphology and structure, which will affect the properties of the obtained films.

In this work, the effect of the degree of substitution in the CMC polymer matrix on the synthesis of silver nanoparticles was studied. The resulting nanoparticles and films were tested for antioxidant and antimicrobial properties and the morphology of the nanoparticles was examined using scanning electron microscopy.

## 2. Results and Discussion

When presenting the results, the abbreviations of the sample names are used as follows:

CMC X + Y

where: X—degree of substitution of CMC (0.7; 0.9; 1.2); Y—the modifier used (MEL—melanin; Ag—Ag nanoparticle synthesized film-forming solution).

### 2.1. Thickness and Mechanical Properties

The thickness and mechanical properties of the films analyzed are shown in Table 1. In general, in this study the addition of melanin had no significant effect on the thickness of the films (*p* < 0.05). The same observations have been made previously for CMC with a degree of substitution of 0.7 modified with different amounts of melanin [38], and for films based on whey protein concentrate/isolate [39] or for alginate [29]. Barely different observations have been made for films based on agar [40], gelatin [41], carrageenan [42] or polybutylene adipate terephthalate (PBAT) [43]. For these polymer matrices, melanin increased the thickness of the films.

Similarly, the addition of silver nanoparticles had no significant effect on film thickness. This observation is supported by the results of Łopusiewicz et al. on the effect of silver nanoparticles on alginate-based films [29].

No statistical differences in tensile strength (TS) were observed between films made from CMC with different degrees of substitution. Additionally, the addition of melanin did not change TS. Similar results were observed for other polymers, but an increase in TS values was observed as the amount of melanin in the film volume increased [29,38]. For samples containing silver nanoparticles, the TS value decreased, but individual films with different degrees of substitution were not statistically different. Similarly, Shankar and Rhim [44] observed a decrease in TS value when silver nanoparticles were added to agar films. Similar observations were made for lactic acid-based films [45,46]. An opposite relationship was found for alginate films modified with silver nanoparticles synthesized using melanin. For these films, TS increased during the presence of silver nanoparticles [29].

Neither the degree of substitution nor the addition of melanin had a significant effect on the elongation at break (EB) value. This stands in contrast to the results presented in the literature for melanin-modified alginate films, where for an analogous melanin concentration there was an increase in EB compared to the pure film control [29]. In another study, a decrease in EB was observed when melanin was added to CMC-based films with a degree of substitution of 0.7, but this study used melanin isolated from mushroom [38]. It also showed the opposite effect to the melanin from watermelon used by Łopusiewicz et al. to modify alginate films, because when added to the alginate matrix, watermelon melanin increased EB values significantly. It is suggested by some researchers that both mechanical parameters may be melanin-dependent in the form that melanin in small amounts increases these values until an inflection point is reached, after which a decrease is observed [42,43,47].

The addition of silver nanoparticles led to a significant, approximately 5-fold increase in EB values. This stands in total opposition to the correlations observed for alginate [29] and polylactic acid (PLA) [46] (decrease in EB), or agar (no change in EB) [48]. On the other hand, for agar films an increase in EB values was also found [44], similarly to what was observed for PLA-based films [45], but the increases were not as significant as in this study.

### 2.2. UV-Barrier Properties

**Figure 1:**
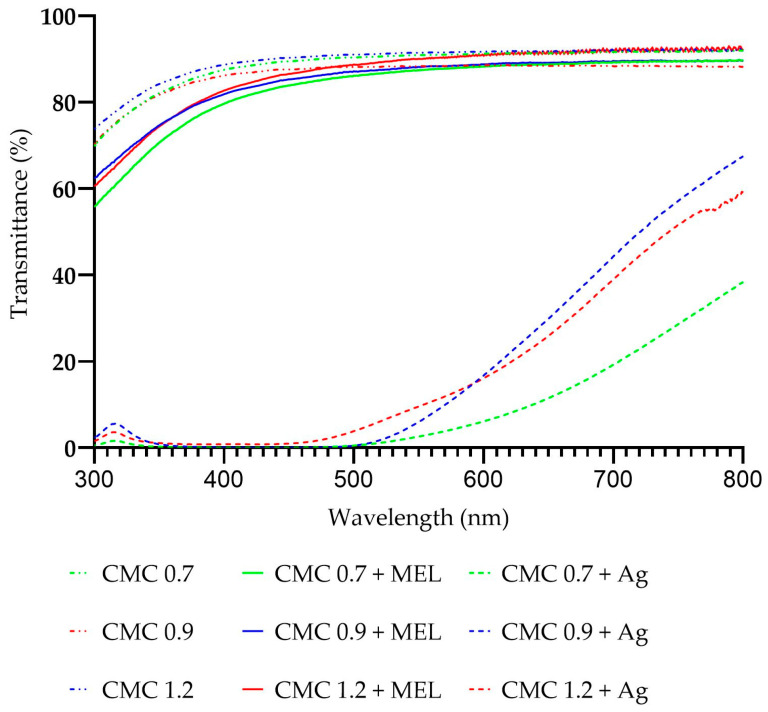
UV–Vis spectra of unmodified, melanin-modified and AgNP-modified CMC films.

In order to evaluate the barrier properties against UV–Vis radiation, spectrophotometric analyses of the obtained films were carried out. Figure 1 shows the UV–Vis spectra of unmodified films, melanin-modified films, and AgNP-modified CMC films. As can be seen, the addition of melanin resulted in improved barrier properties against near-UV and UV radiation. Melanin is well known to have a protective function against UV radiation in animals and plants but also microorganisms, among others [49]. The increase in UV–Vis barrier properties is noticeable but moderate due to the low melanin content of the films and the small thickness of the films. This has been previously demonstrated for melanin-modified CMC-based films [38]. All samples showed similar barrier properties against UV–Vis radiation regardless of the degree of CMC substitution used. Similar light transmission properties have also been described for such melanin-modified polymer matrices as gelatin [41], agar [40], carrageenan [42], alginate [29], cellulose [47], PBAT [43] or chitosan [50].

The addition of silver nanoparticles resulted in a significant reduction in visible light transmittance and almost complete blocking of UV radiation. This property is important from the point of view of packaging products containing photodegradation-sensitive compounds, or in general products sensitive to prolonged contact with sunlight. This is in line with reports regarding the modification with silver nanoparticles of such polymers as agar, alginate or chitosan [29,40,44,51]. In the case of films containing silver nanoparticles, the effect of the degree of substitution on the transparency of visible light through the films is evident. A CMC-based film sample with a degree of substitution of 0.7 modified with melanin and silver nanoparticles shows significantly higher visible radiation blocking properties than CMC 0.9 and CMC 1.2 samples modified with the same additives.

### 2.3. FT-IR Analysis

**Figure 2:**
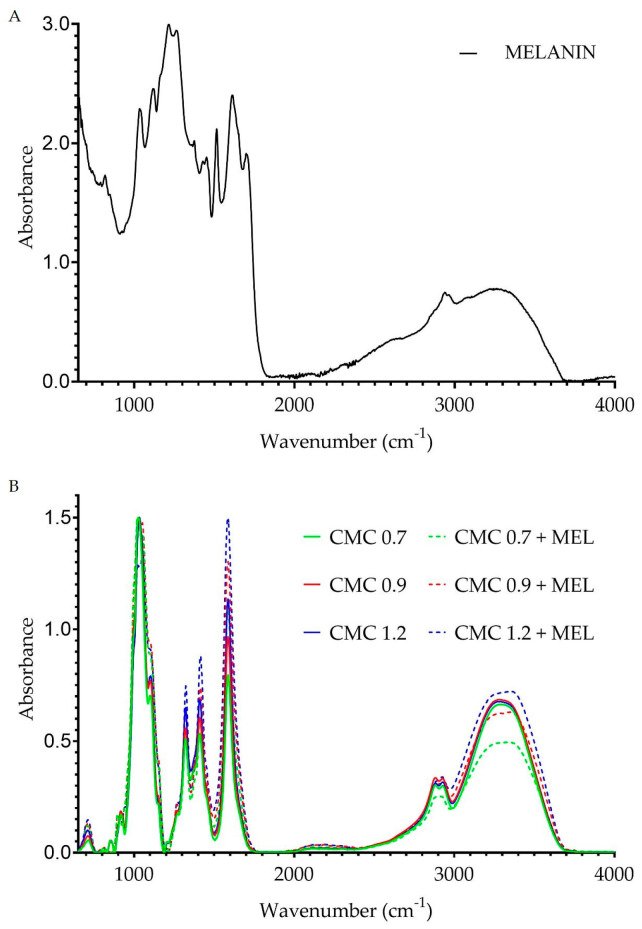
FT-IR spectra of pure melanin (**A**) and unmodified and melanin-modified CMC films (**B**).

FT-IR analysis was carried out to examine the changes in chemical bonds among the samples. Figure 2B summarizes the FT-IR spectra of the unmodified and melanin-modified films while Figure 2A presents the FT-IR spectra of pure melanin as a reference. Both the degree of substitution and the addition of melanin affected the intensity of detection of some of the bonds present in the polymer structure. The intensity of the 3700–3000 cm*^−^*^1^ groups is due to the vibrational frequency of the -OH bonds while the peak at around 2940 cm*^−^*^1^ is related to the stretching vibrations of the C-H bonds [52,53,54]. The peak at 1590 cm*^−^*^1^ corresponds to asymmetric stretching vibrations of -CO bonds and -COO- groups [52,53,54], while 1410 cm*^−^*^1^ corresponds to symmetric stretching vibrations of -CO in the -COO group [52,55] and, further, 1319 cm*^−^*^1^ corresponds to bending vibrations of -OH bonds and 1057 cm*^−^*^1^ corresponds to stretching vibrations of -C-O-C- bonds [54]. The peak at 905 cm*^−^*^1^ is associated with the detection of β-glucosidic bonds between sugar units [56]. The effect of both the degree of CMC substitution [52] and the addition of melanin [29,41,42,43,57] on the intensity of chemical bond detection has already been reported in the literature.

**Figure 3:**
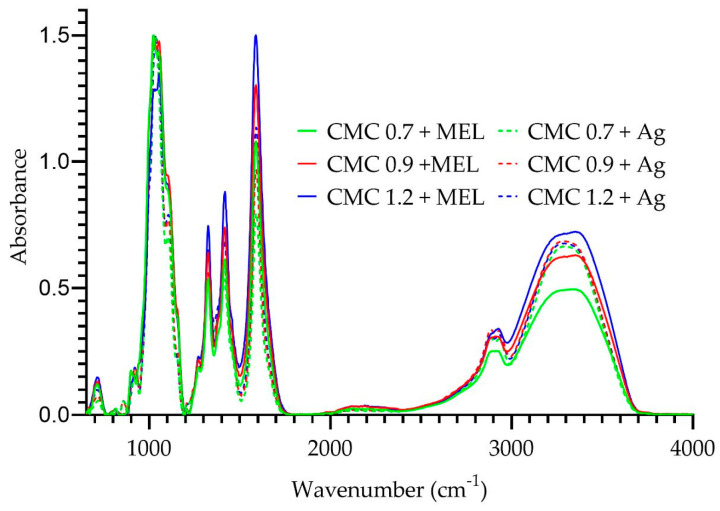
FT-IR spectra of melanin-modified and AgNP-modified CMC films.

Figure 3 shows the changes in the FT-IR spectra of CMC + MEL films after the addition of silver nanoparticles. The addition of silver nanoparticles had a unifying effect on the intensity of the signal in the range of 3700–2800 cm*^−^*^1^. This could potentially indicate the involvement of bonds characteristic of this signal in the in situ synthesis of metal nanoparticles. In the case of peaks in the 1600–600 cm*^−^*^1^ range, changes in signal intensity were observed. The presence of silver nanoparticles caused minor shifts in the absorbance maxima, but no formation of new bonds or disappearance of existing ones was observed. Such an effect of silver nanoparticles stands in agreement with reports for the modification with silver nanoparticles of agar or alginate [29,44], but on the other hand there are also reports on the effect of silver nanoparticles not only on the intensity of bonds but also on their formation and disappearance [51].

### 2.4. SEM Analysis

**Figure 4:**
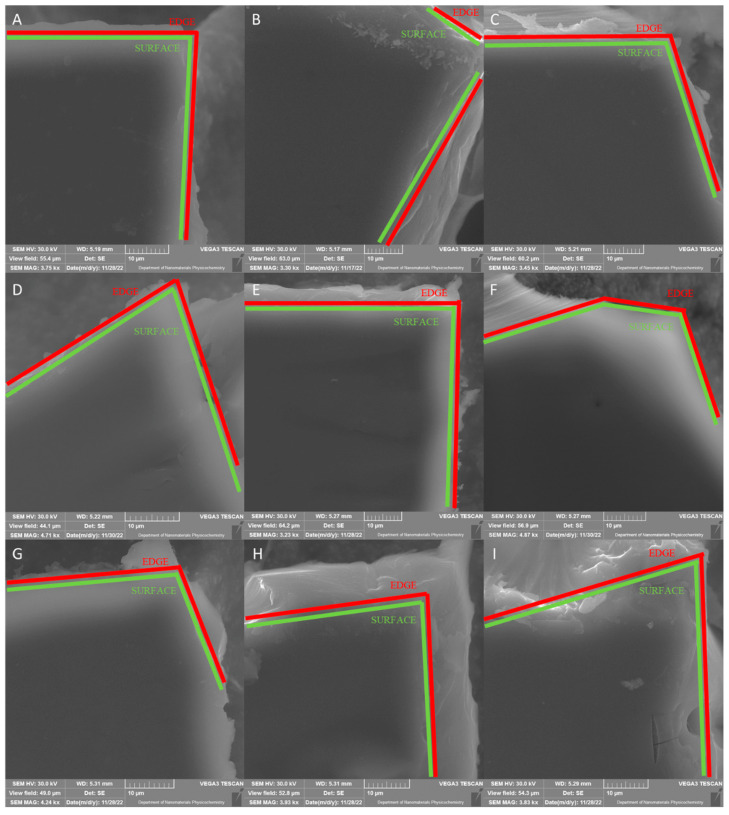
SEM images of CMC 0.7 (**A**), CMC 0.9 (**B**), CMC 1.2 (**C**), CMC 0.7 + MEL (**D**), CMC 0.9 + MEL (**E**), CMC 1.2 + MEL (**F**), CMC 0.7 + Ag (**G**), CMC 0.9 + Ag (**H**), CMC 1.2 + Ag (**I**) of obtained samples.

**Figure 5:**
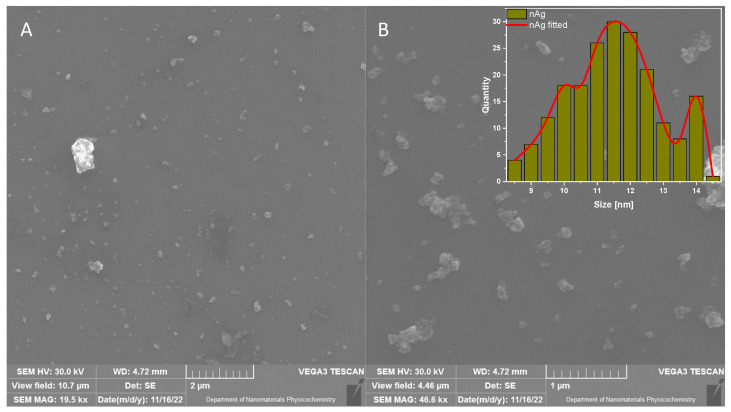
SEM images of silver particles in lower (**A**) and higher (**B**) magnification and (**B**) size distribution.

The morphology of CMC-based films is analyzed by scanning electron microscopy (SEM), and images are presented in Figure 4A–I. The films composed of sodium carboxymethylcellulose (CMC 0.7, CMC 0.9, CMC 1.2—Figure 4A–C) exhibited smooth and homogeneous morphology. It can also be seen that there are no significant differences between films. The addition of melanin to CMC does not change the films’ morphology significantly; the surface of the films is smooth and homogeneous (Figure 4D–F). SEM images of CMC 0.7 + Ag, CMC 0.9 + Ag and CMC 1.2 + Ag are displayed in Figure 4G–I. Here, no notable differences in surface morphology are observed in comparison to CMC and CMC + MEL samples. Additionally, Figure 5 shows SEM images of reference silver nanoparticles (nAg). The histogram of the size distribution of nAg is presented as an inset in Figure 5B. Silver nanoparticles with a size of about 8.5–14.5 nm are observed. The obtained data are in agreement with the literature [58].

### 2.5. XRD Analysis

**Figure 6:**
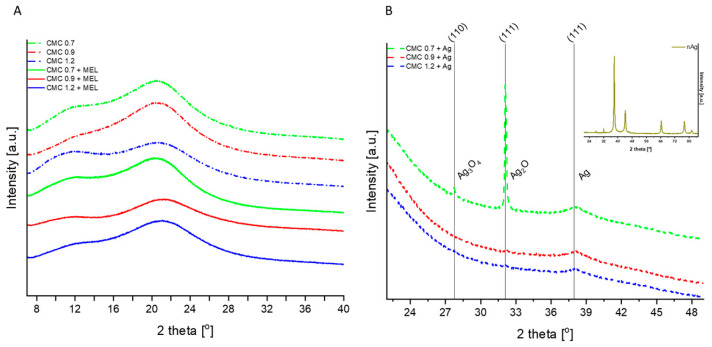
XRD patterns of CMC X and CMC X + MEL, where X is the degree of substitution (**A**), CMC X + Ag and inset of Ag nanoparticles (**B**).

XRD patterns of CMC 0.7, CMC 0.9, CMC 1.2, CMC 0.7 + MEL, CMC 0.9 + MEL and CMC 1.2 + MEL are presented in Figure 6A. Here, no significant difference is detected between CMC and CMC MEL films. For all samples, the broad peaks are observed at 2*θ* values of ~11.8 and ~20.6° which are characteristic of CMC and indicate the amorphous nature of CMC [59]. Moreover, the differences between the degree of substitution and addition of melanin are not observed [60]. XRD patterns of CMC + MEL films modified with nAg (CMC 0.7 + Ag, CMC 0.9 + Ag and CMC 1.2 + Ag) are displayed in Figure 6B. The inset in Figure 6B presents the diffractogram of silver nanoparticles (nAg) and exhibits three peaks at 27.7, 32.1 and 37.9° which correspond to silver (II) and (III) oxide (Ag_3_O_4_; the reference card no: 04-005-4329), silver (I) oxide (Ag_2_O; the reference card no: 01-078-5868) and silver (Ag; the reference card no: 04-014-0266), respectively. The patterns of CMC 0.7 + Ag, CMC 0.9 + Ag and CMC 1.2 + Ag show reflections corresponding to both CMC and nAg phases. It indicates that silver nanoparticles were successfully doped in CMC MEL films. XRD patterns are consistent with SEM results [61,62]. With an increasing degree of substitution in CMC the dispersion of nanoparticles also increases. Due to better dispersion in CMC 1.2 + Ag, nanoparticles are less visible in XRD spectra, however, the presence of nanoparticles is still confirmed. Obtained results are consistent with the literature [63].

### 2.6. Color

**Figure 7:**
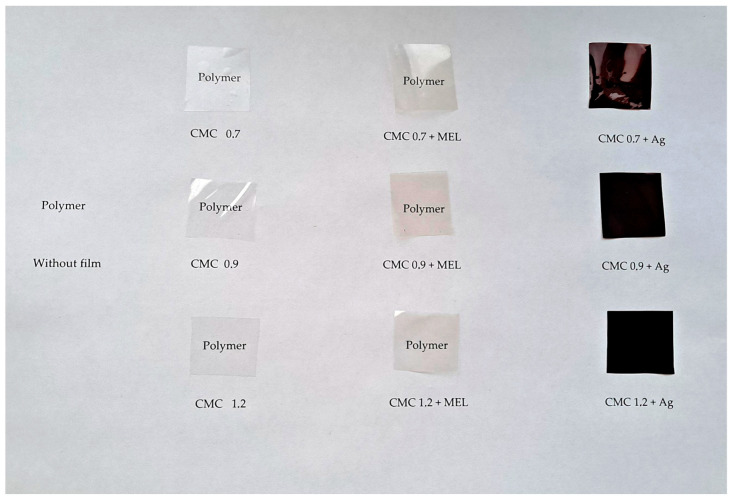
The visual appearance of neat and modified CMC films.

To study the effect of silver nanoparticles synthesized with melanin and the influence of the degree of CMC substitution on the quality of these metallic nanoparticles, chromatic parameter analysis of the film samples was performed. Table 2 shows the chromatic parameters, total color change (ΔE) and yellowing index (YI) of the tested films, while Figure 7 shows the appearance and transparency of the tested films. Neat CMC films, without any additives, did not differ in chromatic parameters, regardless of the degree of substitution. The addition of melanin caused a slight decrease in the L* parameter (the brightness of the films decreased) to a significant degree. However, it did not affect the a* and b* parameters and the opacity of the films. Neither did YI change significantly. Additionally observable was a slight decrease in the transmittance value at 660 nm (T660), which indicates a decrease in the transparency of the films for visible light. For the T280 parameter (UV-blocking properties), there were no significant changes when melanin was added to the films. Nevertheless, the total color change (ΔE) was greater than 1, which is considered to be a difference noticeable to the human eye [64]. Similar relationships, conditioned by the amount of melanin used, were observed for such polymer matrices as gelatin [41], agar [40], alginate [29], carrageenan [42], chitosan [50] or PBAT [43].

The presence of silver nanoparticles resulted in a huge decrease in the L* parameter, and moreover, the degree of substitution had a significant effect on this. As the degree of substitution increased, the brightness of the samples also increased. Likewise, YI as well as the a* and b* parameters (the contribution of red and yellow) significantly increased as the degree of substitution increased. At the same time, the opacity of the films decreased and remained at a similar level regardless of the degree of substitution. The transparency of the films for visible light decreased several times and was lowest for CMC films with a degree of substitution of 0.7 modified with melanin and silver nanoparticles. The transmittance for UV light was 4.51%, 7.65%, 7.98% for substitution degrees 0.7, 0.9, 1.2, respectively. This effect of silver nanoparticles on the color of the films is in agreement with other observations made for alginate [29] or agar [23,44], among others.

### 2.7. Antioxidant Properties

In order to investigate antioxidant properties, the ability to scavenge ABTS and DPPH free radicals was examined and the results are shown in Table 3. For DPPH radicals, although no significant differences were seen between neat films with different degrees of substitution, when melanin was added to the films there was about a two-fold increase in the ability to scavenge free radicals, and the melanin-containing samples themselves were also significantly different after both 20 min and 24 h. Similarly, the free radical scavenging capacity for ABTS^+·^ increased more than 3 times when melanin was added to the films. The antioxidant properties of melanin-modified films have already been demonstrated in numerous publications and are fairly well established [29,38,39,40,41,42,47,50,64].

Silver nanoparticles had an inhibitory effect on both DPPH and ABTS free radical scavenging which resulted in a significant decrease in the measured values for DPPH both after 20 min and after 24 h (below values measured for unmodified films). For ABTS, more than a 2-fold reduction was observed after AgNP addition. The same relationship was previously observed for alginate-based films subjected to similar modifications [29] or for agar films modified with resorcinol and AgNPs [23].

### 2.8. Antimicrobial Properties

To test the antimicrobial properties of the films, an analysis of the films’ ability to inhibit the growth of microorganisms on solid media was applied, and changes in the optical density of the liquid medium inoculated with selected microorganisms were studied.

For melanin-containing films, no growth inhibition zones were observed for any of the microorganisms tested in growth inhibition tests on the medium. This result stands in line with reports for melanin-modified alginate from the same source (watermelon seeds) [29]. On the other hand, another paper reported antimicrobial activity of CMC-based films modified with melanin from a mushroom (*Agarcus bisporus*) against *Escherichia coli, Staphylococcus aureus* and *Candida albicans* [38]. However, melanin from mushroom did not show antimicrobial activity against *E. coli* and *S. aureus* when used to modify the PLA polymer matrix [64]. Potentially, one could conclude that the antimicrobial activity of melanin-modified films is influenced by both the source from which the melanin is derived and the polymer matrix in which it is incorporated, as well as interactions between the two.

Films modified with the addition of silver nanoparticles exhibited antimicrobial activity as zones of inhibition of microbial growth around the disks of the films. This effect was observable for all nanoparticle film samples regardless of the CMC substitution level. Nevertheless, due to the very high solubility of CMC in aqueous environments, the films on the medium were spilling over, which led to the formation of irregular shapes of inhibition zones and made their measurement difficult. This simple and quick test allowed us to confirm the antimicrobial activity of the developed films, but further analysis was needed to determine it more precisely. Representative images of the films on the media and the zones of growth inhibition are presented in the Table S1 (Supplementary Materials).

In order to further determine the antimicrobial properties, analyses of the change in optical density (OD) of liquid cultures of microorganisms in the presence of fragments of the tested films were performed. Since neither CMC films with different degrees of substitution nor melanin-modified films showed antimicrobial properties, one of the films from each group, that is, CMC 1.2 and CMC 1.2 + MEL, was selected as a control for this study.

**Figure 8:**
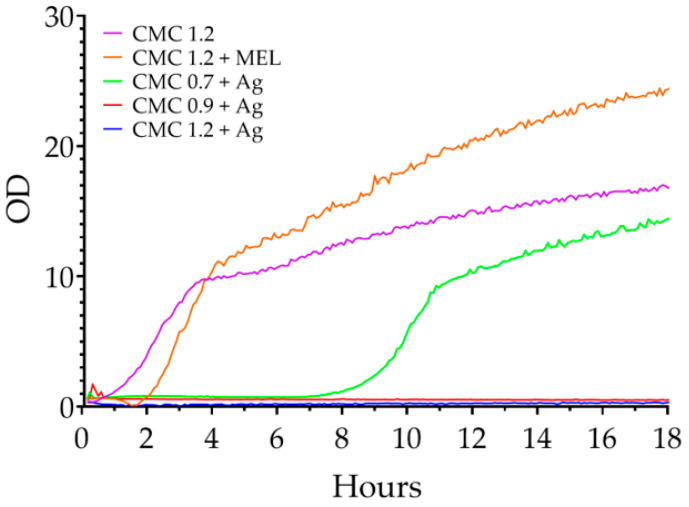
Changes in the optical density of liquid *E. coli* cultures in the presence of the films tested.

The results of the test for *E. coli* are shown in Figure 8. For the control (CMC 1.2), a logarithmic growth phase was observed from the beginning to 3 h, followed by a slower growth moving towards equilibrium from 3 h to the end of the test. The melanin-containing control began a phase of logarithmic growth after 90 min and lasted for 3 h after which slower growth began, lasting until the end of the test. The CMC 0.7 + Ag sample did not start the growth phase until around the 7th hour, and after another 3 it went into a slower growth phase. One suspects that for the first 7 h the nanoparticles present in the sample had an inhibitory effect on the proliferation of microorganisms, but after this time there was a consumption of all the nanoparticles present in the sample (for example, by binding to genetic material in the microorganisms—a genotoxic effect of the nanoparticles [65]). Consequently, those microorganisms that managed to survive for that time could begin to multiply successfully. For samples with degrees of substitution of 0.9 and 1.2 modified with silver nanoparticles, no changes were observed in the optical density of the cultures over the duration of the test.

**Figure 9:**
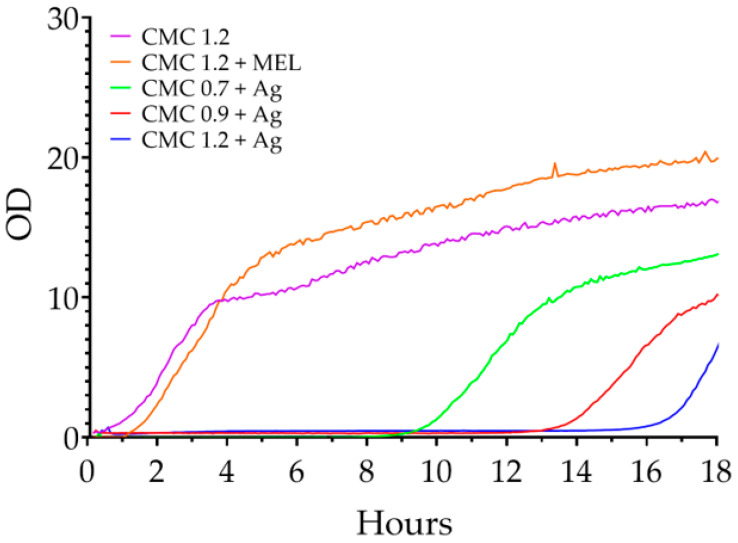
Changes in the optical density of liquid *S. aureus* cultures in the presence of the films tested.

Figure 9 includes changes in the optical density of liquid *S. aureus* cultures in the presence of the tested films. As early as around 1 h, the cultures containing the control samples began to enter the logarithmic growth phase, and at around 4 h they transitioned to slow growth. The transition from the resting phase (phase-Lag) to the logarithmic growth phase occurred after 9, 13 and 17 h for CMC 0.7 + Ag, CMC 0.9 + Ag and CMC 1.2 + Ag, respectively. This clearly indicates a relationship between the degree of substitution and the quality/quantity of nanoparticles obtained in the film.

**Figure 10:**
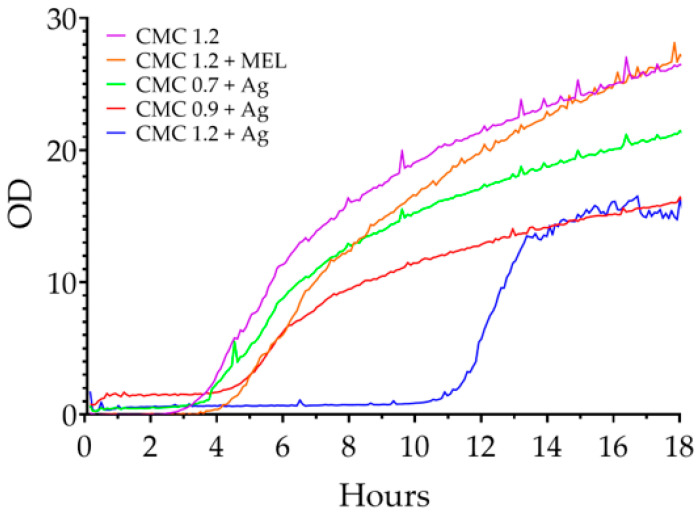
Changes in the optical density of liquid *C. albicans* cultures in the presence of the films tested.

Changes in the optical density of liquid cultures of *C. albicans* are shown in Figure 10. Here, the onset of logarithmic growth occurred between 3 and 4 h for all samples, except CMC 1.2 + Ag for which this phase began around the 11th hour. Again, this indicates the influence of the degree of substitution on the nanoparticles obtained.

**Figure 11:**
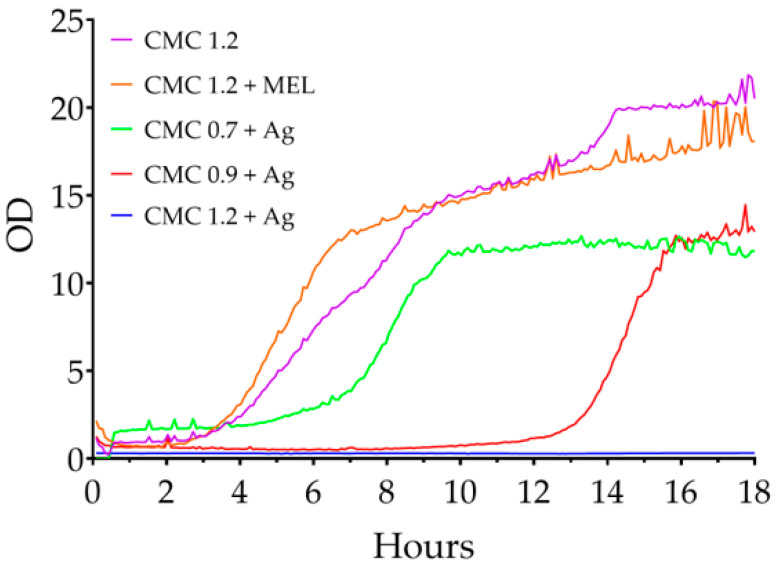
Changes in the optical density of liquid *P. aeruginosa* cultures in the presence of the films tested.

The effect of the films tested on the proliferation of *P. aeruginosa* is shown in Figure 11. Again, the relationship between the degree of substitution and antimicrobial properties can be seen. Cultures containing control films were the first to enter the logarithmic growth phase, followed after 4 h by CMC 0.7 + Ag film, and 5 h later by CMC 0.9 + Ag film samples. No OD changes were observed in the culture containing CMC 1.2 + Ag film during the test.

**Figure 12:**
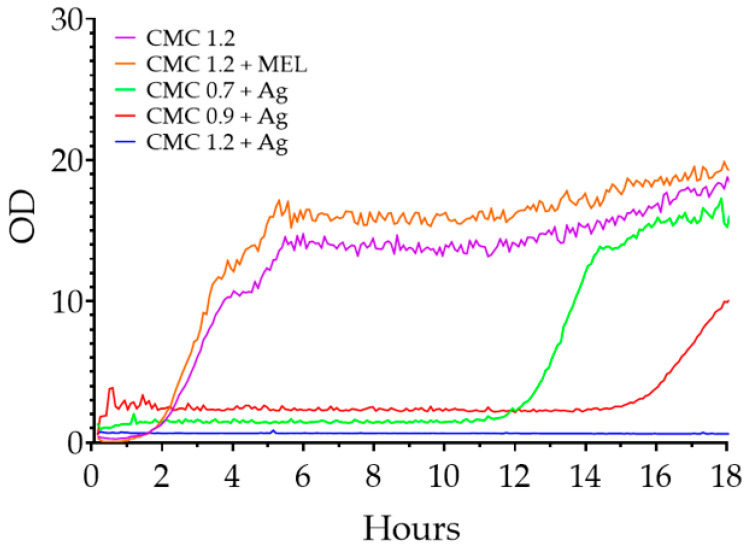
Changes in the optical density of liquid *B. cereus* cultures in the presence of the films tested.

Figure 12 shows the changes in optical density of *B. cereus* cultures. As in the above cases, the fastest (after 2 h) beginning of logarithmic multiplication of bacteria was observed in the presence of control samples. After another 10 h, microorganisms cultured in the presence of the CMC 0.7 + Ag sample entered this phase, after another 4 h for the CMC 0.9 + Ag sample, while for the CMC 1.2 + Ag sample no start of proliferation was recorded over the course of the study.

The differences in the effects of the same films on different microorganisms may be explained by differences in the structure of their cell walls and cell membranes, the rate of division and the size of the genetic material. As was mentioned regarding AgNPs, once they are released from the polymer matrix, they affect microorganisms through the formation of pores in membranes (interaction with sulfur contained in membrane proteins) and interaction with genetic material. Therefore, the same amounts of nanoparticles can affect different microorganisms with different intensities.

As mentioned in the SEM imaging section, the silver nanoparticles obtained with melanin showed sizes between 8.5 and 14.5 nm on average. These sizes are in line with literature data on the antimicrobial properties of silver nanoparticles [23,30,44,46,66,67]. As was proven by Hebeish et al., as the degree of CMC substitution increases, the dispersion of silver nanoparticles increases [37], and consequently they exhibit an ever-increasing lethal effect against microorganisms. The effects of silver nanoparticles on microorganisms such as *E. coli* [23,44,48,51], *S. aureus* [45,46], *Bacillus* sp. and *Klebsiella pneumoniae* [51] or *Listeria monocytogenes* [23,44,48] have been reported in the literature. Here, it is shown that the antimicrobial properties of CMC polymer matrix films modified with silver nanoparticles synthesized in situ depend on the degree of CMC substitution.

## 3. Materials and Methods

### 3.1. Materials and Reagents

1,1-diphenyl-2-(2,4,6-trinitrophenyl) hydrazyl (DPPH), 2,2′-azino-bis (3-ethylbenzothiazoline-6-acid) sulfonic acid (ABTS), potassium persulfate, sodium carboxymethyl cellulose (Mw = 250,000; DS = 0.7, 0.9, 1.2) were purchased from Sigma-Aldrich (Darmstad, Germany). Glycerol, ammonia water, chloroform, ethanol and methanol were from Chempur (Piekary Śląskie, Poland). Peptone water and MacConkey medium were from Scharlau Chemie (Barcelona, Spain). Silver (III) nitrate was from POCh (Gliwice, Poland). Chapman’s medium and plate count agar were from Merck (Dramstadt, Germany). All reagents were of analytical grade. The microorganisms used to evaluate the antimicrobial properties of the films were obtained from the American Type Culture Collection (ATCC, Manassas, VA, USA). The strains used were *Escherichia coli* ATCC8739 and *Staphylococcus aureus* ATCC12600, *Candida albicans* ATCC14053, *Pseudomonas aeruginosa* ATCC 39327, *Bacillus cereus* ATCC13061.

### 3.2. Preparation of Films

Melanin was isolated and purified from fresh watermelon (Citrullus lanatus) seeds as previously described [39]. To obtain melanin solutions, melanin was added to distilled water in the amount required to reach a concentration of 0.25% (*w*/*w*). An alkaline environment was then created to allow better melanin dissolution by adding ammonia water in an amount of 1 mL for every 200 mL of melanin solution. The prepared solutions were incubated overnight on an orbital shaker at a stirring speed of 200 rpm and a temperature of 60 °C.

This was followed by dividing the solutions into equal parts in separate bottles (200 mL of solution per bottle) and dividing them into 3 groups. CMC with a degree of substitution equal to 0.7, 0.9 or 1.2 was added to 1/3 of the bottles in an amount of 4 g so as to obtain concentrations of 2% (*w*/*v*). Dissolution of the added polymer was carried out using a water bath and a temperature of 40 °C with continuous stirring. Glycerol, acting as a plasticizer, was added to the prepared film-forming solutions in an amount of 0.3 g for every 1 g of CMC used and then stirred on a magnetic stirrer for 10 min. The final prepared solutions were poured onto square polystyrene plates (120 mm × 120 mm) at 35 g of solution per plate and dried at 40 °C for 48 h. As a control, melanin-free films were prepared using the same procedures. All films were prepared in 4 replicates.

Films with silver nanoparticles (AgNPs) were prepared according to the above procedure with an additional step of adding an aqueous silver nitrate solution to the film-forming solutions. To the solutions with dissolved CMC placed on a magnetic stirrer (stirring speed of 1000 rpm), an aqueous silver nitrate solution was slowly added with a pipette until a final concentration of 10 mM was reached in the film-forming solution. The solutions were then placed in a water bath at 87 °C for 1 h until they reached a dark brown color indicative of AgNP synthesis. The films were then cooled to room temperature, plasticizer was added and the films were poured onto plates as described above. All films were prepared in 4 replicates. The films obtained are presented in Table 4.

### 3.3. Preparation of Nanoparticles

In addition, nanoparticles alone were prepared as a reference for nanoparticles synthesized in the presence of CMC polymer matrix. For this purpose, melanin solutions (0.25% *w*/*w*) were prepared as described above and nanoparticles were synthesized in the presence of melanin alone. An aqueous solution of silver nitrate was slowly added to the melanin solutions until a concentration of 10 mM was reached, stirring all the time, and then the solutions were placed in a water bath at 87 °C for 1 h. After this time, the solutions were cooled to room temperature, transferred to Falcon tubes and centrifuged (6000 rpm for 20 min) to separate the synthesized particles from the aqueous fraction. The aqueous fraction was poured off and the resulting precipitate was dried at 40 °C overnight, then kept in Falcon-type tubes until analysis.

### 3.4. Characterization of Biocomposite Films

#### 3.4.1. Thickness and Mechanical Properties

Film thickness was measured using an electronic thickness gauge (Dial Thickness Gauge 7301, Mitoyuto Corporation, Kawasaki, Kangagawa, Japan) with an accuracy of 0.001 mm. Each sample was measured 10 times at randomly selected points and the results were averaged.

The tensile strength and elongation at break of the films were assessed using a Zwick/Roell 2.5 Z static testing machine (Ulm, Germany). The tensile clamp spacing was 25 mm and the head travel speed was 100 mm/min.

#### 3.4.2. Spectral Analysis of Films

UV–Vis spectra of films were measured using a Thermo Scientific (Waltham, MA, USA) Evolution 220 UV–Vis spectrophotometer. Strips of films, with a surface area matching the quartz cuvette (5.5 cm × 1 cm), were placed along with a cuvette in the apparatus and spectra were recorded over a wavelength range of 300–800 nm, with a resolution of 1 nm.

The chemical composition of the obtained films was also evaluated using a Perkin Elmer Spectrum 100 FT-IR spectrophotometer (Waltham, MA, USA). Pieces of the films were measured directly, in ATR mode (32 scans per sample), and spectra were recorded over a wavelength range of 650–4000 cm^−1^, with a resolution of 1 cm^−1^. Melanin spectra, as a reference, were obtained in the same way.

#### 3.4.3. Structure and Morphology Analysis of Films

The structure and morphology of obtained materials were analyzed via scanning electron microscopy (SEM) (VEGA3 Tescan). The crystallographic information of samples was obtained via X-ray diffraction (XRD), performed using an AERIS PANalytical X-ray diffractometer with Cu-Kα radiation.

#### 3.4.4. Color Analysis of Films

The effect of melanin and silver nanoparticles on the color of the obtained films was assessed using a colorimeter (CR-5, Konica Minolta, Tokyo, Japan). Illuminant D65 was used. Each sample was tested 10 times, at randomly selected points. The results (mean ± standard deviation) were expressed as L*, a* and b* parameters. In addition, color difference (ΔE) and yellowing index (YI) were calculated for comparison with unmodified alginate films, using the following equations:

ΔE = [(L_standard_ − L_sample_)^2^ + (a_standard_ − a_sample_)^2^ + (b_standard_ − b_sample_)]^0.5^

YI = 142.86b·L^−1^

#### 3.4.5. Antioxidant Properties of Films

The antioxidant properties of the films were determined by examining the scavenging of DPPH and ABTS radicals. For this purpose, 100 mg of each film was placed in 5 mL of 0.01 mM DPPH in methanol solution and incubated in the dark for 20 min and 24 h, respectively. DPPH solution without the addition of the tested films was used as a blank control. Absorbance of the solutions (associated with DPPH radical scavenging) was measured at 517 nm using a 96-well plate and plate reader. Free radical scavenging activity was calculated from the following formula:

Free radical scavenging activity (%) = A_control_
*−* A_sample_*/*A_control_ × 100

where A_sample_ is the absorbance of the DPPH solution with the addition of the tested films, and Ac_ontrol_ is the absorbance of the blank DPPH solution.

ABTS free radicals were induced by mixing 5 mL of 7 mM ABTS with 2.45 mL of potassium persulfate and leaving overnight in the dark at room temperature. The solution was then diluted using ethanol until the absorbance of the final solution was 0.7. Then, 100 mg of each of the test films was placed in Falcon tubes together with 10 mL of ABTS^+^ solution and incubated in the dark for 6 min. After this time, the absorbances of the solutions were measured at 734 nm. The free radical scavenging capacity of ABTS^+·^ was calculated from the same formula as for DPPH. ABTS^+·^ solution without the addition of films was used as a control.

#### 3.4.6. Antimicrobial Properties

In order to determine the antimicrobial properties of the prepared films, the growth inhibition of microorganisms on plate count agar (PCA) was measured in the presence of the tested films’ disks. For this purpose, one-day cultures of *Escherichia coli, Staphylococcus aureus, Candida albicans, Pseudomonas aeruginosa* and *Bacillus cereus* were added to separate tubes with sterile peptone water until they reached an optical density of 0.5 on the McFarland scale. The suspensions of microorganisms (200 uL each) were then applied to separate Petri dishes (90 mm in diameter) with PCA, spread with disposable strokes and 2 disks of each film (20 mm in diameter) were applied. After a 24 h incubation at 37 °C, the size of the inhibition zones was measured.

In addition, the antimicrobial activity of the films was tested using bioreactors by placing 1 × 1 cm fragments in Falcon-type tubes with a 20 mL suspension of microorganisms at an optical density of 0.5 on the McFarland scale. The bioreactor (Biosan Ltd. bioreactor RTS-1, Lithuania) was set to maintain a constant temperature of 37 °C, the tubes were rotated at 500 rpm and the direction of rotation changed every 2 s. In addition, it examined changes in the optical density of the suspensions at 5 min intervals. After an overnight incubation, the results obtained from the change in optical density during culture were recorded. As a control, films CMC 1.2 and CMC 1.2 + MEL were used.

### 3.5. Statistical Analysis

All analyses were carried out at least in triplicate. Statistical analysis was performed using Statistica version 13 software (StatSoft Poland, Krakow, Poland). Differences between means were determined by analysis of variance (ANOVA) followed by Fisher’s post hoc LSD test at a significance threshold of *p* < 0.05.

## 4. Conclusions

This paper presents a process for obtaining CMC-based films with different degrees of substitution (0.7, 0.9 and 1.2) modified with melanin and silver nanoparticles. The silver nanoparticles were synthesized in situ using melanin as a nanoparticle stabilizer. The aim of the study was to determine what effect the degree of substitution of the CMC polymer matrix has on the synthesis and properties of the nanoparticles. The degree of substitution had no significant effect on the mechanical and antioxidant properties of the films. What is noticeable is its effect on color (films with a degree of substitution of 0.7 with silver nanoparticles were clearly the darkest, followed by films with degrees of substitution of 0.9, while films of 1.2 were the least dark). Similarly, the barrier properties against UV–Vis had a substitution degree-dependent character—the lower the substitution degree, the stronger the barrier properties. The presence of silver nanoparticles affected the changes in the FT-IR spectrum of the films by altering the intensity of some of the bonds. The degree of substitution was also found to affect the antimicrobial properties of the films. As the degree of substitution increased, increasingly better antimicrobial activity was found (inhibition of microbial proliferation was longer the higher the degree of substitution of films modified with silver nanoparticles). It can be concluded that the obtained CMC films modified with melanin and silver nanoparticles could find potential applications in food packaging and biomedical applications.

## Figures and Tables

**Table 1:** Thickness and mechanical characteristics of unmodified and modified CMC films.

Sample	Tensile Strength (MPa)	Elongation at Break (%)	Thickness (mm)
CMC 0.7	34.6 ± 17.1 ^a,b,c,d^	5.4 ± 3.4 ^a^	0.064 ± 0.017 ^a,b^
CMC 0.9	34.9 ± 10.4 ^b,c,d^	6.6 ± 5.0 ^a^	0.067 ± 0.013 ^a,b^
CMC 1.2	25.2 ± 11.1 ^a,b,c^	7.2 ± 6.1 ^a^	0.081 ± 0.026 ^a^
CMC 0.7 + MEL	45.8 ± 17.0 ^d^	3.5 ± 1.3 ^a^	0.048 ± 0.016 ^b^
CMC 0.9 + MEL	47.9 ± 14.4 ^d^	4.4 ± 1.4 ^a^	0.055 ± 0.012 ^a,b^
CMC 1.2 + MEL	39.4 ± 9.52 ^c,d^	5.0 ± 2.7 ^a^	0.053 ± 0.018 ^a,b^
CMC 0.7 + Ag	18.8 ± 5.46 ^a,b^	30.6 ± 13.0 ^b^	0.079 ± 0.014 ^a^
CMC 0.9 + Ag	15.5 ± 3.05 ^a^	31.3 ± 13.8 ^b^	0.061 ± 0.019 ^a,b^
CMC 1.2 + Ag	24.6 ± 8.41 ^a,b,c^	30.8 ± 12.9 ^b^	0.058 ± 0.013 ^a,b^

Values are means ± standard deviation. Means with different lowercase letters are significantly different at *p* < 0.05.

**Table 2:** Color (L*, a*, b*), total color difference (ΔE), yellowness index (YI) and transmittance of unmodified and modified CMC films.

Sample	L*	a*	b*	ΔE	YI	Opacity	T280 (%)	T660 (%)
CMC 0.7	78.30 ± 7.01 ^b^	−0.57 ± 0.07 ^a^	13.32 ± 0.77 ^a^	Used as standard	23.89 ± 1.02 ^a^	7.56 ± 0.16 ^a^	82.54 ± 1.31 ^b^	91.39 ± 1.34 ^a^
CMC 0.9	77.82 ± 1.48 ^b^	−0.52 ± 0.14 ^a^	12.55 ± 1.72 ^a^	Used as standard	25.23 ± 2.08 ^a^	7.52 ± 0.08 ^a^	89.75 ± 1.29 ^d^	88.41 ± 1.64 ^a^
CMC 1.2	88.52 ± 2.04 ^e^	−0.69 ± 0.03 ^a^	14.35 ± 0.43 ^a^	Used as standard	23.22 ± 0.17 ^a^	7.50 ± 0.16 ^a^	90.61 ± 0.94 ^d^	91.75 ± 1.75 ^a^
CMC 0.7 + MEL	70.57 ± 5.24 ^a^	−0.33 ± 0.05 ^a^	14.69 ± 0.63 ^a^	9.22 ± 1.50 ^d^	29.43 ± 1.06 ^a^	7.49 ± 0.11 ^a^	79.53 ± 1.73 ^b,c^	88.87 ± 1.48 ^a^
CMC 0.9 + MEL	73.72 ± 1.62 ^a^	−0.34 ± 0.03 ^a^	14.64 ± 0.16 ^a^	4.95 ± 1.20 ^c^	28.33 ± 0.34 ^a^	7.42 ± 0.11 ^a^	81.20 ± 1.63 ^b^	89.23 ± 1.45 ^a^
CMC 1.2 + MEL	71.08 ± 2.12 ^a,b^	−0.33 ± 0.02 ^a^	14.50 ± 0.29 ^a^	17.45 ± 2.13 ^e^	29.25 ± 0.31 ^a^	7.41 ± 0.07 ^a^	76.44 ± 1.27 ^c^	91.56 ± 1.39 ^a^
CMC 0.7 + Ag	2.87 ± 0.76 ^c^	7.07 ± 2.24 ^b^	3.79 ± 1.08 ^b^	76.42 ± 0.59 ^a,b^	185.86 ± 11.27 ^b^	3.96 ± 0.15 ^b,c^	4.51 ± 0.58 ^a^	12.85 ± 1.82 ^c^
CMC 0.9 + Ag	5.59 ± 1.30 ^c^	16.16 ± 3.14 ^c^	8.54 ± 2.27 ^c^	74.94 ± 0.74 ^a^	218.07 ± 7.33 ^c^	3.89 ± 0.11 ^b^	7.65 ± 0.84 ^a^	32.67 ± 1.13 ^b^
CMC 1.2 + Ag	13.45 ± 1.11 ^d^	18.51 ± 0.80 ^d^	21.49 ± 2.18 ^d^	77.85 ± 0.66 ^b^	227.99 ± 4.36 ^d^	4.14 ± 0.10 ^c^	7.98 ± 0.75 ^a^	28.31 ± 1.48 ^b^

Values are means ± standard deviation. Means with different lowercase letters are significantly different at *p* < 0.05.

**Table 3:** Free radical (DPPH and ABTS) scavenging activity of unmodified and modified CMC films.

Sample	DPPH 20 min (%)	DPPH 24 h (%)	ABTS (%)
CMC 0.7	14.17 ± 0.14 ^b,c^	19.29 ± 0.24 ^a^	24.74 ± 0.56 ^a^
CMC 0.9	14.44 ± 0.23 ^c^	20.52 ± 0.95 ^a^	25.72 ± 0.93 ^a^
CMC 1.2	14.03 ± 0.19 ^b^	19.64 ± 0.67 ^a^	24.49 ± 0.49 ^a^
CMC 0.7 + MEL	23.54 ± 0.22 ^e^	54.47 ± 0.53 ^c^	81.28 ± 1.07 ^c^
CMC 0.9 + MEL	24.32 ± 0.41 ^d^	53.85 ± 0.80 ^c^	84.73 ± 1.28 ^d^
CMC 1.2 + MEL	26.04 ± 0.36 ^f^	54.41 ± 0.48 ^c^	88.94 ± 1.14 ^e^
CMC 0.7 + Ag	8.87 ± 0.30 ^a^	12.02 ± 0.47 ^b^	34.76 ± 1.47 ^b^
CMC 0.9 + Ag	8.73 ± 0.24 ^a^	12.47 ± 0.71 ^b^	35.53 ± 1.73 ^b^
CMC 1.2 + Ag	8.92 ± 0.29 ^a^	12.67 ± 0.59 ^b^	34.94 ± 1.82 ^b^

Values are means ± standard deviation. Means with different lowercase letters are significantly different at *p* < 0.05.

**Table 4:** Summary of films obtained.

Sample	CMC Degree of Substitution	Presence of Melanin	Presence of AgNPs
CMC 0.7	0.7	No	No
CMC 0.9	0.9	No	No
CMC 1.2	1.2	No	No
CMC 0.7 + MEL	0.7	Yes	No
CMC 0.9 + MEL	0.9	Yes	No
CMC 1.2 + MEL	1.2	Yes	No
CMC 0.7 + Ag	0.7	Yes	Yes
CMC 0.9 + Ag	0.9	Yes	Yes
CMC 1.2 + Ag	1.2	Yes	Yes

## Data Availability

Not applicable.

## Supplementary Materials

The supporting information can be downloaded at: https://www.mdpi.com/article/10.3390/ijms232415560/s1.
