# Supplementary material for: Bioactive Carboxymethyl Cellulose (CMC)-Based Films Modified with Melanin and Silver Nanoparticles (AgNPs)—The Effect of the Degree of CMC Substitution on the In Situ Synthesis of AgNPs and Films’ Functional Properties
Source: Int J Mol Sci. 2022 Dec 8;23(24):15560. doi: 10.3390/ijms232415560 (PMC9779376; doi:10.3390/ijms232415560)
Supplement: Supplementary file 1 [file ijms-23-15560-s001.zip › ijms-2075437-supplementary.pdf]

Sample	SD 0.7	SD 0.9	SD 1.2	Microorganism
CMC + MEL	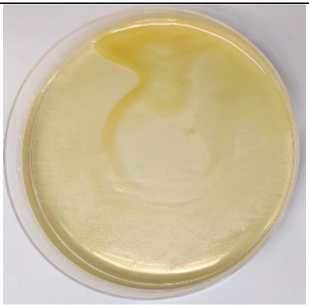	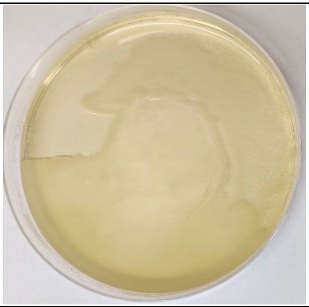	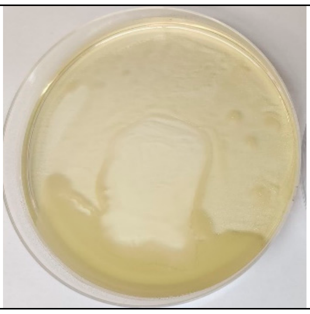	*Candida albicans*
CMC + Ag	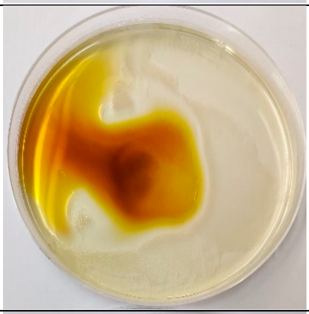	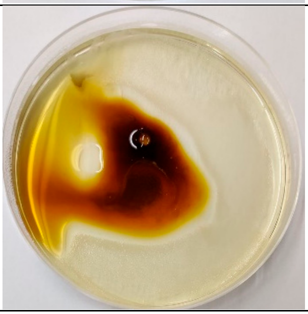	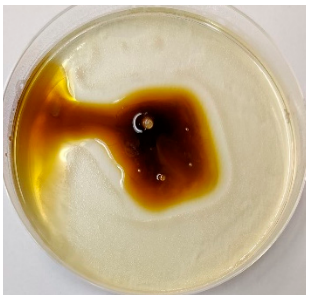	
CMC + MEL	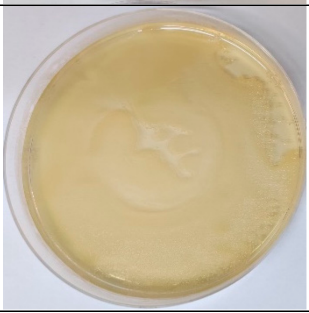	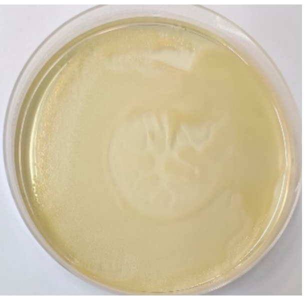	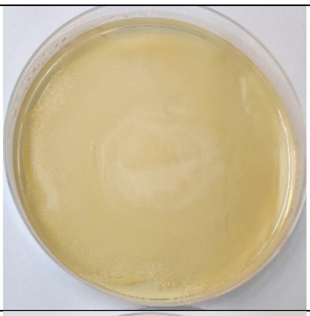	*Staphylococcus aureus*
CMC + Ag	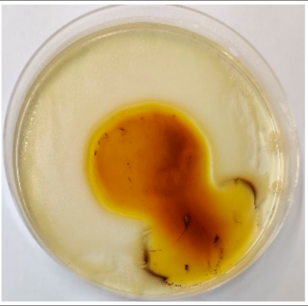	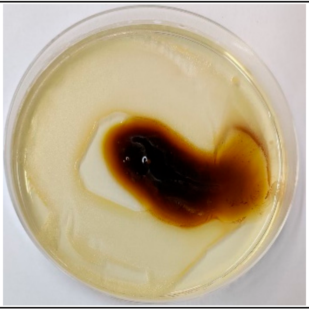	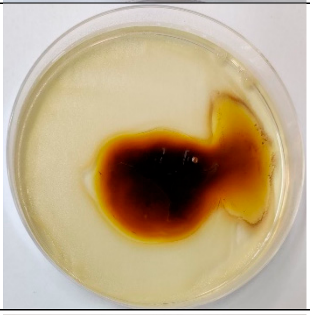	
CMC + MEL	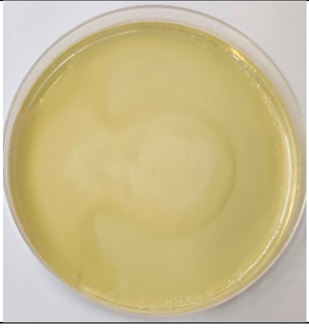	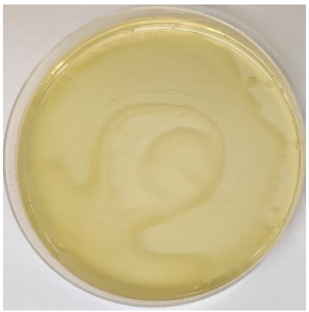	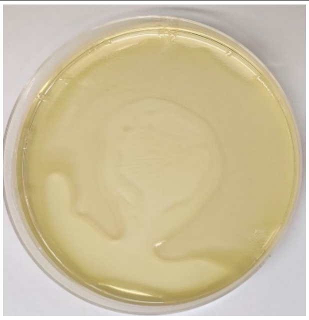	*Escherichia coli*
CMC + Ag	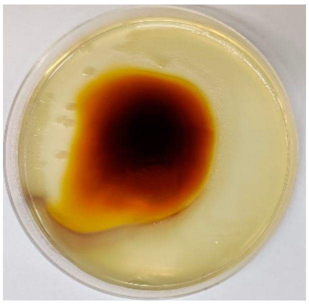	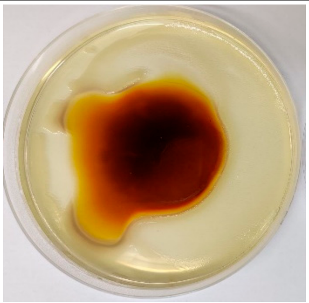	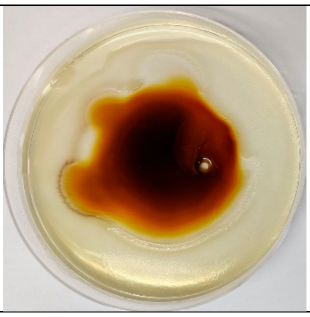	

CMC + MEL	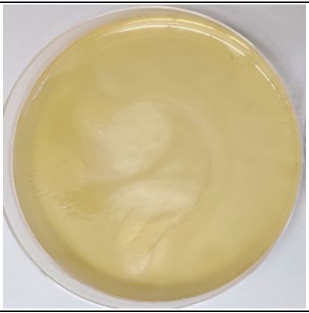	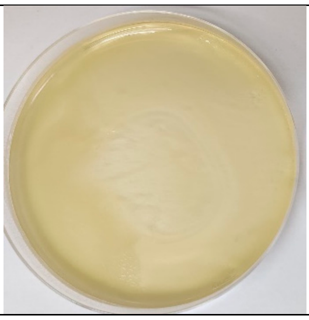	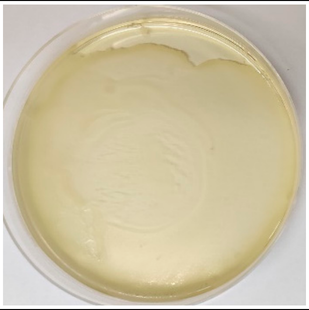	*Bacillus cereus*
CMC + Ag	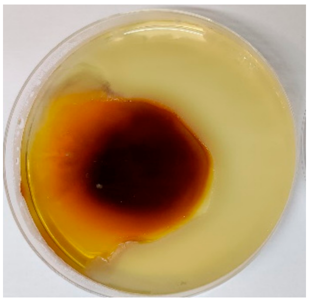	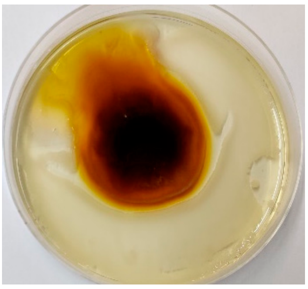	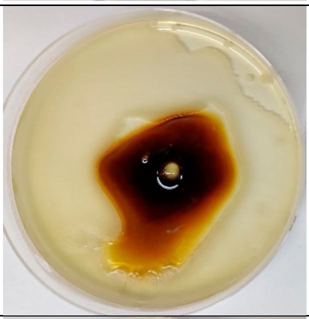	
CMC + MEL	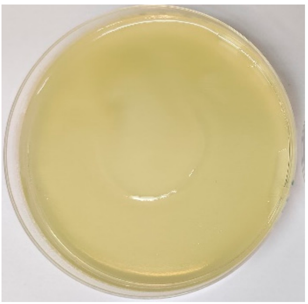	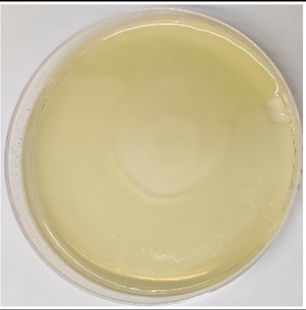	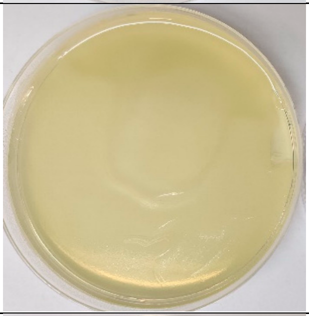	*Pseudomonas aeruginosa*
CMC + Ag	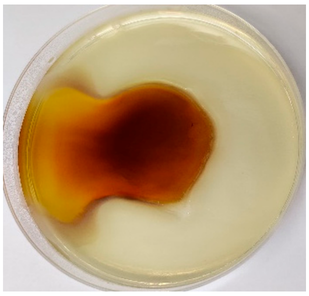	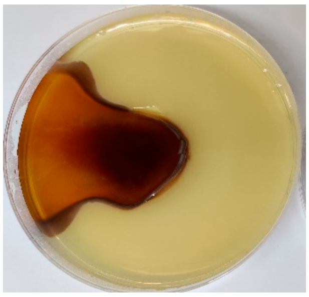	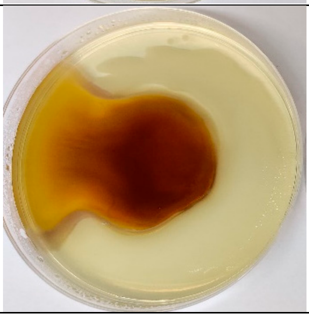	

Table S1. Representative photos of growth inhibition zones of melanin-modified and AgNPs-modified films
